# Evaluating the Role of Nutrient Competition in *Debaryomyces hansenii* Biocontrol Activity Against Spoilage Molds in the Meat Industry

**DOI:** 10.3390/jof11040242

**Published:** 2025-03-22

**Authors:** Helena Chacón-Navarrete, Ignacio García-Álvarez de Toledo, José Ramos, Francisco Javier Ruiz-Castilla

**Affiliations:** 1 Department of Agricultural Chemistry, Edaphology and Microbiology, University of Córdoba, Campus de Rabanales, 14014 Córdoba, Spain; b62chnah@uco.es (H.C.-N.); ignacio.garcia@irta.cat (I.G.-Á.d.T.); mi1raruj@uco.es (J.R.); 2 Food Safety and Functionality Programme, IRTA, Finca Camps I Armet S/N, 17121 Monells, Spain

**Keywords:** *Debaryomyces hansenii*, biocontrol, nutrient competition, molds, food safety

## Abstract

The rejection of chemical preservatives reflects the growing demand for natural and safe products. This concern has spurred scientific interest in yeasts as biocontrol agents, given their antagonistic activity against undesired fungi, which is one of the main problems associated with preservative reduction. *Debaryomyces hansenii* is a non-conventional yeast that has shown great potential for inhibiting filamentous fungi in the food industry. This study investigated the role of nutrient competition in the biocontrol activity of *D. hansenii* against unwanted molds. Potentially pathogenic molds from spoiled food were isolated using different media and identified using Sanger sequencing. The inhibitory effects of different autochthonous *D. hansenii* strains under varying nutrient conditions were assessed against isolated molds using semipermeable membranes. Inhibition activity was measured by assessing mycelial expansion and spore production using image software analysis and classical cell counting using a Neubauer chamber. The results indicated that *D. hansenii* effectively inhibited mold growth and sporulation, with the autochthonous strains LR2 and SRF1 showing higher inhibitory activity than the control strain CBS767. The effectiveness of inhibition varied with the yeast–mold combination, highlighting the need for a species-specific analysis. Nutrient competition plays a complementary role in *D. hansenii* biocontrol but does not directly impact overall inhibition. This suggests that other mechanisms, such as direct cell interactions or metabolite production, may be crucial. These findings enhance our understanding of the potential of *D. hansenii* as a natural preservative and advance biocontrol methods for food safety.

## 1. Introduction

The rejection of chemical preservatives has been trending among consumers in recent years, reflecting a growing demand for attractive, healthy, and safe products that maintain their quality using natural methods. This consumer concern has also resonated within the scientific community, as evidenced by numerous studies highlighting the potential health risks associated with the overuse of chemical preservatives [1,2,3]. Over the past few decades, researchers and industries have shown interest in using yeast as a potential biocontrol agent. These microorganisms have the potential to partially replace artificial preservatives, particularly due to their antagonistic activity against undesired fungi [4,5,6]. Several yeast species have demonstrated this characteristic behavior, including *Pichia guilliermondii, Candida intermedia*, *Kluyveromyces marxianus*, *Saccharomyces cerevisiae*, *Yarrowia lipolytica,* and *Debaryomyces hansenii* [7]. *D. hansenii* has emerged as a species of particular interest for inhibiting undesired filamentous fungi in the food industry [8,9,10]. Its significance extends beyond its antifungal properties, as its presence can add new characteristics and value to food products, resulting in a combination of attributes that are highly desirable in the food industry [11]. An additional advantage of *D. hansenii* is its safety profile for human consumption, as evidenced by its inclusion in the European Food Safety Agency (EFSA) Qualified Presumption of Safety (QPS) list, further supporting its potential as a natural preservative in food products.

*D. hansenii* is a hemiascomycetous yeast belonging to the order Saccharomycetaceae and is considered a non-conventional yeast. This microorganism is part of the CTG clade, characterized by its unique codon usage, where CUG encodes serine instead of the more common leucine [12]. This distinctive trait places *D. hansenii* phylogenetically closer to species like *Candida albicans* rather than *S. cerevisiae*. The evolutionary distance between *D. hansenii* and other yeasts explains many of its unique characteristics. These include high gene density and significant heterogeneity among different strains. Notably, *D. hansenii* exhibits variability in chromosome numbers, ranging from 4 to 10, across different strains [13,14,15,16]. These characteristics contribute to the genetic diversity and adaptability of strains, making them an interesting subject for research in comparative genomics and evolutionary biology within the yeast kingdom.

Furthermore, *D. hansenii* is a potential game changer in the meat industry, as its presence contributes to the aroma profile, texture, and taste of cured meat by hydrolyzing sarcoplasmic proteins [17] and producing exopeptidases [18]. In addition to these effects, this yeast has been widely studied due to its antifungal capacity previously mentioned against various filamentous fungi from genera like *Penicillium, Aspergillus*, and *Mucor* [19,20,21]. This biocontrol activity has been explained based on different mechanisms, such as protective biofilm production, enzyme excretion, nutrient and space competition, and volatile compound generation [22,23]. This capacity is an exceptional and natural means of preserving the organoleptic characteristics of cured meat products while ensuring safety for consumption and economic profitability [19,24,25].

When researching fungal interactions, nutrient competition emerges as a critical factor influencing fungal growth. As key players in nutrient cycling, fungi often compete for limited resources, which significantly impacts their growth, survival, and ecological roles. Understanding the effects of nutrient competition on fungi is crucial for applications in biotechnology, agriculture, and ecosystem management. Fungal growth is notably influenced by the spatial distribution of nutrients, with fungi tending to grow more in nutrient-rich areas and forming reproductive structures in response to their environment’s nutrient status [26,27,28,29]. This interaction is particularly interesting when considering the dynamics between yeast and filamentous fungi. Soil yeasts generally exhibit stronger antagonistic effects on filamentous fungi compared to phyllosphere yeasts, with this antagonism being species-specific and not dependent on the type of filamentous fungus [30]. Certain soil yeasts, such as *Candida subhashii*, *Metschnikowia pulcherrima*, and *D. hansenii* [10,11,30], are especially effective in suppressing filamentous fungi. These yeasts can assimilate and oxidize a wider range of carbohydrates and organic acids, indicating a competitive advantage in nutrient utilization [30].

As evident from the literature, there is an extensive body of research regarding the mechanisms involved in the antifungal capability of *D. hansenii*, which proposes this yeast as a candidate for use as a starter culture. The use of starter cultures is one of the most innovative techniques in the field of fermented foods. These cultures offer several advantages, including the homogenization of production batches, enrichment and enhancement of the native microbiota in these foods, control over the development profile, and, when using microorganisms that act as biocontrol systems, protection against external contamination, a major concern in the industry. Some autochthonous *D. hansenii* strains have already been used as starter cultures on a small scale, achieving promising results in the meat industry [11,31].

Following the described relevance of the nutrient competition mechanism, it is no wonder that it is one of the primary mechanisms mentioned by authors when discussing fungal inhibition exerted by *D. hansenii*. However, there is a notable lack of specific information describing its action in this context. While many articles suggest that nutrient competition may be responsible for fungal inhibition by *D. hansenii*, detailed information on this mechanism is scarce. Several studies mention this potential mechanism without providing in-depth explanations [32,33,34], and those that do inquire into this mechanism are scarce and do not provide specific information about its importance in the overall fungal inhibition mechanism [35,36]. Understanding the specific role of *D. hansenii* in nutrient competition with filamentous fungi is key to fully understanding the biocontrol functionality of this yeast.

To further elucidate the mechanisms by which *D. hansenii* inhibits potentially pathogenic fungi, we conducted a series of experiments focused on investigating the role of nutrient competition in the inhibition process and assigning a quantitative percentage of the action of this mechanism in the overall antifungal capability of *D. hansenii*. Our approach involved isolating and identifying several filamentous fungi from spoiled food. We assessed the inhibitory effect of *D. hansenii* strains on these isolated fungi and to determine whether the inhibition was specifically due to nutrient competition, we applied conditions of both nutrient excess and shortage to evaluate the consistency of the results across different environmental conditions. The findings of this study provide new, specific data on the nutrient competition mechanism of *D. hansenii*, which has been poorly understood until now. These results not only enhance our understanding of the antifungal properties of *D. hansenii* but also open potential avenues for its application as a natural preservative in the food industry, particularly in preserving dry-cured meat products like Iberian Pork Loin, potentially leading to more effective and natural food preservation methods in the future.

## 2. Materials and Methods

### 2.1. Strains and Culture Conditions

Three different *D. hansenii* strains were used in this study. The strains SRF1 and LR2 are autochthonous *D. hansenii strains* originally isolated from Iberian dry meat products in Valle de los Pedroches [37]. Additionally, the wild-type strain CBS767 from the Fungal Biodiversity Center in the Netherlands [38] was used as a comparative control. Four mold isolates from the genera *Penicillium*, *Mucor*, and *Aspergillus* were also studied. Yeasts were cultured on Yeast Peptone Dextrose Agar media (YPD) containing 1% yeast extract, 2% peptone, and 2% dextrose (D-glucose) (*w*/*v*). Solid media were prepared by adding 2% (*w*/*v*) agar. *D. hansenii* SRF1, LR2, and CBS767 strains were incubated at 26 °C for two days to obtain starting cultures. For molds, Potato Dextrose Agar media (PDA) was used with incubation at 28 °C for at least five days to allow for sporulation. The different media used in this work across different experiments included Potato Dextrose Agar (PDA), Potato Dextrose Broth (PDB), Sabouraud Chloramphenicol Agar (SC), De Man–Rogosa–Sharpe Calcium Carbonate Agar (MRS), Plate Count Agar (PCA), and Yeast Maltose Broth (YMB). The specific compositions were as follows: PDA and PDB were prepared using a commercial mixture from OXOID, with a composition of 4 g/L potato extract, 20 g/L dextrose, and 15 g/L agar for PDA. SC was prepared using a commercial mixture from Scharlau, with a composition of 40 g/L D (+)-glucose, 5 g/L casein peptone, 5 g/L meat peptone, 15 g/L agar, and 0.05 g/L chloramphenicol. MRS media was prepared using a commercial mixture from OXOID, composed of 10 g/L peptone, 8 g/L meat extract powder, 4 g/L yeast extract, 20 g/L D (+)-glucose, 1 mL sorbitan monooleate, 2 g/L dipotassium phosphate, 5 g/L sodium acetate trihydrate, 2 g/L triammonium citrate, 0.2 g/L magnesium sulfate heptahydrate, 0.05 g/L manganese sulfate tetrahydrate, and 10 g/L agar, with the final mixture supplemented with 10 g/L CaCO_3_. PCA was prepared using a commercial mixture from Oxoid with a composition of 5 g/L tryptone, 2.5 g/L yeast extract, 1 g/L D (+)-glucose, and 9 g/L agar. Finally, YMB was prepared from a commercial mixture of Millipore containing 5 g/L peptic digest of animal tissue, 3 g/L yeast extract, 3 g/L malt extract, and 10 g/L dextrose. In this work, 20 g/L agar was added for solid media usage.

### 2.2. Isolation of Filamentous Fungi from Contaminated Samples

Several samples from Iberian Pork Loins with low preservative conditions as well as commercial ones (Table 1) were used to isolate fungal species that could detriment the final quality of the product. The samples used were stored under optimal conditions, but the detrimental preservatives led to contamination, which was the object of the study. Meat samples were treated as follows: the loins were cut with sterile scalpels, and approximately 10 g of each condition was obtained. The samples were then placed in individually marked Stomacher bags containing 45 mL of buffered peptone water and homogenized. Once homogenized, the mixtures were transferred to new tubes.

From the treated samples, serial dilutions were prepared to obtain isolates of the different microorganisms present in the meat using selective media cultures, as described in the section “Strains and culture conditions”. Due to the wide variety of colonies observed in the different cultures, the initial criterion for isolation was phenotypic differentiation. Each isolate was named following a code consisting of three parts: the first letter indicated the type of microorganism being isolated, with three groups identified by the letters “L” for yeasts, “H” for filamentous fungi, and “B” for bacteria; the second part of the name corresponded to the media from which the microorganism was isolated: PDA for Potato Dextrose Agar, SC for Sabouraud Chloramphenicol Agar, PCA for nutritive agar, and MRS for De Man–Rogosa–Sharpe (MRS) Calcium Carbonate Agar. Finally, a number was added to each code according to the order in which it was identified (Table 2).

### 2.3. Identification of Filamentous Fungal Isolates

To identify the genera and species of the different isolates, Sanger sequencing was performed. DNA extraction from the samples was carried out using Chelex extraction based on the protocol described by Conlon et al. [39] with minor modifications specified below. Approximately 10 mg of mycelium was scraped off the agar using a sterile scalpel and transferred to an empty Eppendorf tube. The mycelium at this step can be stored at −20 °C before continuing with the extraction. To each mycelium sample, 200 μL of Chelex solution was added along with 10 μL of 20 mg/mL proteinase K. The Chelex solution was kept on a magnetic stirrer to ensure sample homogeneity. The sample was mixed using a vortex, transferred to a tube, and incubated in a thermocycler at 65 °C for 30 min. The samples were then centrifuged at 3300× *g* for 3 min. Finally, 100 μL of the supernatant was transferred to a new tube. The genetic material obtained was amplified using MyTaq™ HS Red DNA Polymerase (Meridian Bioscience, Cincinnati, OH, USA) following the manufacturer’s instructions and using *ITS1-30F*/*ITS1-217R,* as described by Usyk et al. [40].

### 2.4. Fungal Inhibition Assay in the Presence of D. hansenii Strains

The assay was adapted from the methodology described by Medina-Córdova et al. [8], who demonstrated the ability of *D. hansenii* to form biofilms that inhibit the colonization of other microorganisms. Our research group previously conducted a similar assay [10] using different strains of *D. hansenii* and various filamentous fungi distinct from those used in this study.

Precultures of the *D. hansenii* strains SRF1, LR2, and CBS767 were cultivated in YPD broth at 26 °C with agitation at 150 rpm. Cell suspensions of *D. hansenii* were prepared at a concentration of 10^6^ CFU/mL and spread onto YPD agar plates, followed by incubation at 26 °C for 24 h. Spore suspensions of the different molds were prepared by scraping the surface of agar plates and transferring the spores into Falcon tubes containing sterile distilled water, with the spore concentration adjusted to 10^4^ spores/mL. Then, 10 μL of the spore suspension was inoculated at the center of each yeast pre-cultured plate. The co-cultured plates were then incubated for 3 days at 28 °C. Three independent biological replicates were used. The results were quantified by measuring the diameter of the fungal colonies on both the co-cultured plates and the control plates without yeast with the image analyze software Image J 1.53. The percentage of inhibitory activity (IA) was calculated using the formula [(C − T)/C] × 100. In the formula, C (control) is the total area of mycelial expansion in the control plates without yeast, and T (treatment) is the total area of mycelial expansion in the co-cultured plates.

### 2.5. Fungal Inhibition Assay After D. hansenii Interaction. Inhibition Activity Through Semipermeable Membranes

Sterile cellophane membranes were used as semipermeable membranes [41] to test whether the effect of yeast was still active after its removal. Cellophane membranes were placed on YMB agar medium plates to cover the entire contact surface. A 100 μL cell suspension was inoculated onto these plates and spread using a Digralsky handle to cover half of each plate. The inoculated plates were incubated at 26 °C for 3 days. After incubation, the yeast and cellophane were removed using a sterile clamp.

Two possible types of inhibitory activity were investigated to collect the results. One was directly on the contact surface of the yeast, and the other expanded beyond the direct contact zone. To differentiate between these two possible activities, six droplets of spore suspension prepared as described in the section “Fungal inhibition assay in the presence of *D. hansenii* strains” were added to the yeast-free agar plates. Three were in the contacted zone, and the rest were in the other zone. The plates were then incubated for 3 days at 28 °C. Independent biological replicates were used for each experiment. The results were obtained by measuring the diameter of the fungal colonies on both plates with yeast and the control plates without yeast. The IA was calculated using the same formula described in Section 2.4.

#### 2.5.1. Nutrient Competition Mechanism Assay

To check whether the nutrient content of the media had a significant effect on the inhibition rates, the assay was replicated using modified media. To do so, YMB media were prepared by adding 50% more and 50% less of the commercial mix while maintaining the agar proportion at 2% in all cases. Six independent biological replicates were used for the assay. The results were quantified as described in the previous assay and used to compare the inhibition ratios among the different conditions.

#### 2.5.2. Inhibition of the Fungal Reproductive System. Spore Counting

To test all the effects produced by the yeast on mold growth, spore production was quantified. From each Petri dish, a 1 cm diameter piece of agar was taken from the center of the fungal inoculation zone using a sterile metal screw-in tool. The agar piece, together with the spores and mycelium, was transferred to an Eppendorf tube containing 1 mL of sterile distilled water and vortexed for at least 1 min to release the spores from agar. Serial dilutions were then performed, and the concentration of spores per milliliter was quantified using a Neubauer chamber at 40× magnification. A total of six replicates per condition were performed. The IA was calculated following the same formula as described in the section “Materials and Methods”; however, instead of the area of mycelial growth, spore quantification data were used.

### 2.6. Data Quantification and Statistical Analysis

All experiments were performed in triplicate. In the experiments that required area measurements, results were quantified using the software Image J (v.1.53) (open access), the data were analyzed with Microsoft Excel 2016 (Licensed by University of Córdoba, Microsoft Corporation, Redmond, WA, USA) software and the significance of differences between mean values was determined by GraphPad Prism 9 (Licensed by University of Cordoba, Dotmatics, Boston, MA, USA). Representations were performed using Python 3.12.6. The packages panda, numpy and matplotlib were used for data manipulation, numerical operations, and data visualization, respectively.

## 3. Results

### 3.1. Isolation and Identification of Filamentous Fungi from Contaminated Samples

A total of 47 isolates were obtained from the different cultures according to phenotypic characterization. Of these, thirteen were identified as yeasts, twenty-six as bacteria, and the remaining eight as filamentous fungi. Fungal isolates identification information is displayed in Table 2. Data regarding the variability of isolates found under each nutrient condition are presented in Table 3 as the percentage of the number of species found from the total number of isolates.

From the isolated filamentous fungi, four were chosen to continue the inhibition assays, as they had a more negative impact on the overall meat quality due to unpleasant aromas, mycotoxin production, and/or unwanted flavors [42,43,44]. The selected isolates were *Penicillium crustosum* HSC30 (PC), *Penicillium rubens* HPDA10 (PR), *Mucor circinelloides* HYPD3 (MC), and *Aspergillus flavus* HSC31 (AF). In addition to the fungi isolates, an additional species from a previous assay was used as a control: *Aspergillus niger* HCH1 (AN) (Laboratory collection, University of Córdoba, Córdoba, Spain).

### 3.2. Fungal Inhibition Assay in the Presence of D. hansenii Strains

**Figure 1:**
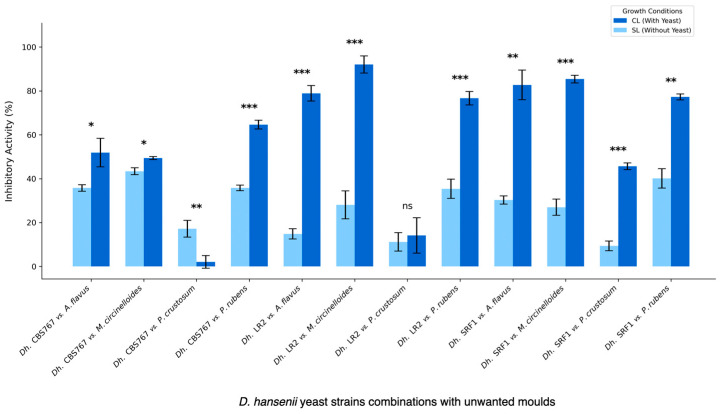
Bar plot representation of inhibitory activities (IAs) obtained in the assays described in the sections “Fungal inhibition assay in the presence of *D. hansenii* strains” and “Nutrient competition mechanism assay”. The different molds tested were *A. flavus* (AF), *M. circinelloides* (MC), *P. crustosum* (PC), and *P. rubens* (PR). The presented data compare the IA percentages obtained under the same nutrient conditions (standard) when confronting molds directly against yeast (CL) or indirectly (SL). Mean values ± standard deviation obtained from at least three independent biological replicates are plotted. Significant differences between each CL-SL yeast–mold combination are indicated by asterisks: * = *p*-value ≤ 0.05, ** = *p*-value ≤ 0.01, *** = *p*-value ≤ 0.001, ns = not significant.

The results shown in Figure 1 represent the effect of *D. hansenii* strains on mycelial growth. The results are presented as the inhibitory action percentage (IA), as described in Section 2.4. Clearly, when *D. hansenii* is in direct contact with the mold, the inhibition becomes significantly higher than the indirect influence in most cases.

**Figure 2:**
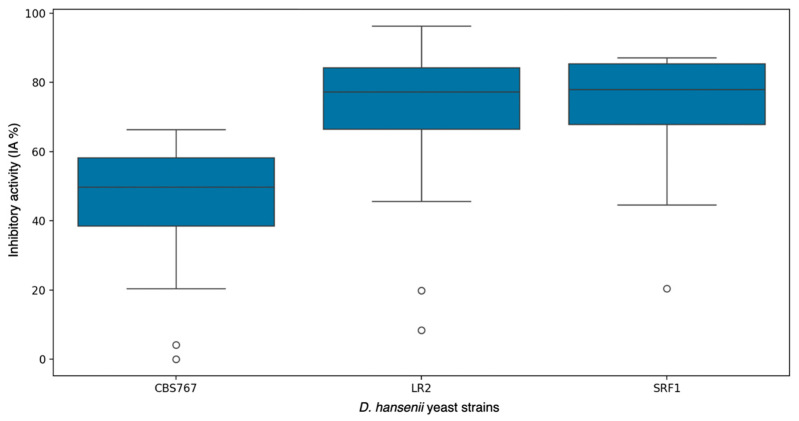
Box plot representation of mean inhibitory activities (IAs) obtained by the *D. hansenii* strains CBS767 (Control), LR2, and SRF1 (autochthonous) in the assay described in the section “Fungal inhibition assay in the presence of *D. hansenii* strains”. The control values of IA are not plotted, as they always correspond to a zero value. The presented IA values for each yeast strain are the means of the IA of all the studied molds.

When analyzing each strain individually and unifying the inhibition results for all studied molds, the data showed no significant differences among them (Figure 2). *D. hansenii* SRF1 showed the highest mean inhibitory activity with 70.59%, closely followed by *D. hansenii* LR2 with 68.07%. Lastly, *D. hansenii* CBS767 showed a mean inhibitory activity of 43.52%. Notably, this strain was the only non-autochthonous strain used in this work.

**Figure 3:**
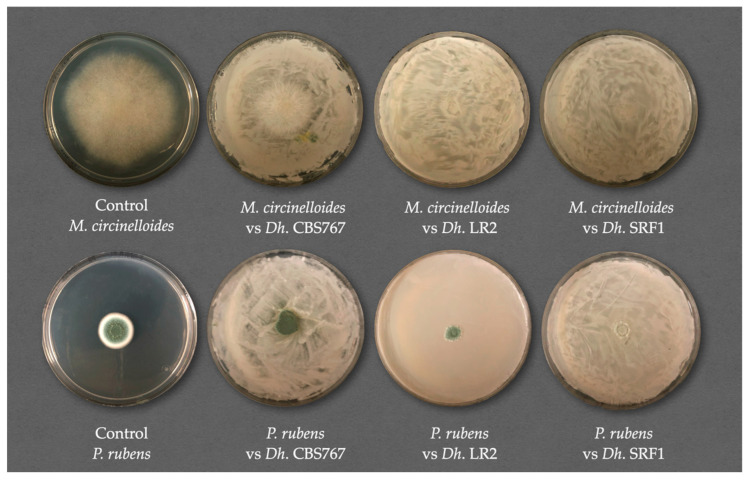
Representative images of the action of *D. hansenii* (*Dh*) strains CBS767, LR2, and SRF1 against the molds *M. circinelloides* (MC) and *P. rubens* (PR). The yeast was incubated for 24 h before inoculating with the mold spores. Images were captured 3 days after mold inoculation.

**Figure 4:**
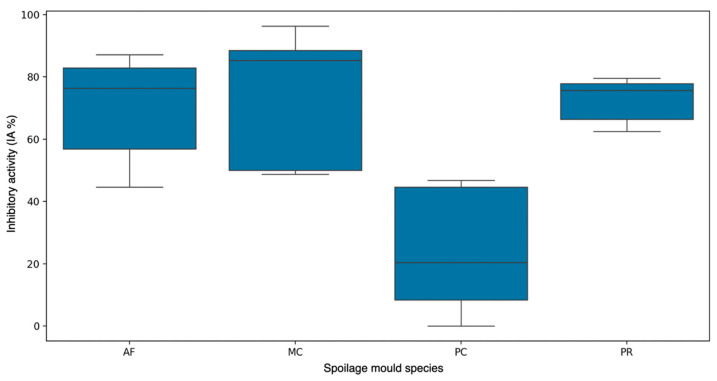
Box plot representation of mean inhibitory activities (IAs) obtained for each studied mold by the *D. hansenii* strains CBS767 (Control), LR2, and SRF1 (autochthonous) in the assay described in the section “Fungal inhibition assay in the presence of *D. hansenii* strains”. The control values of IA are not plotted as they always correspond to a zero value.

As shown in Figure 1, all molds were affected by *D. hansenii*, although to a different extent. Examples of the observed inhibition are shown in Figure 3. MC was the most affected mold overall, with an inhibition of more than 90%. AF and PR showed inhibition of around 70%, and PC showed an average inhibition of about 40% (Figure 4).

### 3.3. Direct Versus Indirect Contact: Antifungal Assessment of D. hansenii Against Various Molds

The results showed that in most cases, the inhibitory activity with direct yeast presence (CL) was higher than that without direct presence (SL). The PC mold was an exception, as SL often showed a higher IA percentage than CL across different yeasts (Figure 1). The effectiveness of inhibition varied significantly depending on the yeast–mold combination, but *D. hansenii* LR2 strain seemed to be the most efficient, especially against AF and MC molds. The SRF1 yeast strain consistently performed better under CL conditions for all mold types. The CBS767 yeast strain showed mixed results, with better performance under SL conditions than under PC conditions.

### 3.4. Inhibition of Molds Post D. hansenii Action: Inhibition Activity Through Semipermeable Membranes

**Figure 5:**
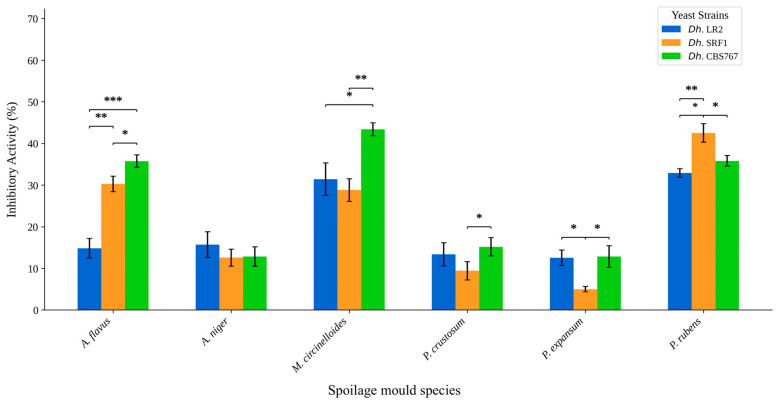
Bar plot representation of mean inhibitory activities (IAs) obtained for each studied mold−yeast interaction in section “Inhibition of mold post *D. hansenii* action: Inhibition activity through semipermeable membranes” Plotted data represents the mean values ± standard deviation obtained from at least three independent biological replicates of standard nutrient concentration condition. Significant differences between each CL-SL yeast−mold combination are plotted with asterisks: * = *p*-value ≤ 0.05, ** = *p*-value ≤ 0.01, *** = *p*-value ≤ 0.001.

**Figure 6:**
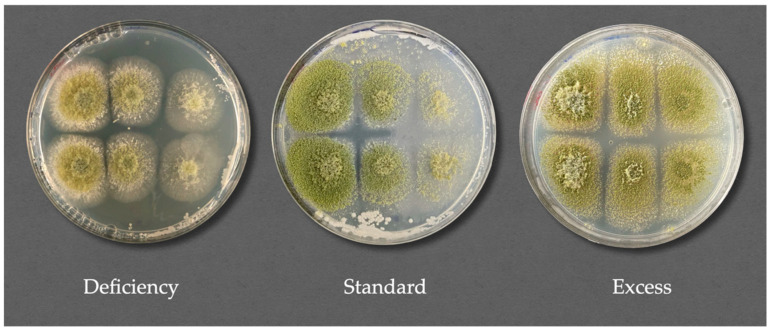
Representative images of the action of *D. hansenii* SRF1 strain against the mold *A. flavus* (AF) under different nutrient conditions: deficiency, standard, and excess. The plates were covered with a cellophane membrane, inoculated only on the right side of the plates, and incubated for 3 days. After incubation, the cellophane membranes were removed, and the molds were inoculated as described in the section “Fungal inhibition assay after *D. hansenii* interaction. Inhibition activity through semipermeable membranes” and incubated for another 3 days. In the images, clear differences can be observed when first looking at the left part of the plates, where there was no yeast, and the right part, where yeast had interacted.

When this effect was studied, the data turned out to be complex; therefore, different approaches were tested to better understand the outcome of the different assays performed. The results showed that in all cases, significant inhibition of mycelial expansion was achieved, although it varied between the mold and yeast strain combinations. In general, the inhibition never surpassed 45%. MC was again the most affected mold by the three strains in the study, with CBS767 being the most effective one with 43.40% inhibition in contrast with the action of SRF1 and LR2 with around 27% and 28%, respectively. Following MC, we found PR with an overall inhibition rate of 30% and AF of 25%. Finally, PC, PE, and AN were the least affected, with inhibition percentages of a maximum of 15% (Figure 5). These results contrast with the macroscopic observation of the different plates, as there were outstanding differences between the treated and untreated portions in the plates (Figure 6).

#### 3.4.1. Nutrient Competition Mechanism Assay

To evaluate the importance of the nutrient competition mechanism in the different experiments, multiple statistical analyses were performed. The experiments in which cellophane membranes were used as semipermeable membranes were replicated with two different nutrient concentrations: an excess of 50% of the overall composition and a shortage of 50% of the overall composition. Therefore, in contrast to the usual formula, the nutrient concentrations in the commercial formula were 50% and 150%, respectively. First, we provided an overview of all the inhibition data collected by a one-way ANOVA, which resulted in a *p*-value of 0.00021 (Table S1), indicating that, in the first approach, the inhibitory activity was significantly influenced by the nutrient concentration modifications. It is remarkable that in a second approach to this collective data through a Tukey test, these significant differences were more specific, as significant differences were found only when comparing the standard condition to the modified ones, but not the modified ones against each other (Table S2). The conditions with fewer nutrients led to an increase in IA compared to the other conditions. To fully understand the complexity of the situation, we will focus on each specific mold studied.

#### 3.4.2. Specific Mold Species Inhibition Under Different Nutrient Concentration Conditions

The results indicated that nutrient conditions significantly affected IA for *Aspergillus* sp. species, but only in specific cases. For AF, the most evident and significant difference was between the shortage and standard conditions, with inhibition values of around 38% and 14%, respectively. In the AN case, significant differences were found between the standard and both modified concentrations. The most particularly evident difference for this species was between the shortage and standard conditions, with inhibition values of around 18% and 27%, respectively. In the case of MC, nutrient conditions did not significantly affect IA, and no significant differences were observed.

*Penicillium* sp. showed diverse results among the studied species; in general, no significant differences were found among the yeast strain inhibition rates. Nutrient conditions significantly affected IA when comparing the nutrient conditions.

For PE and PC, significant differences were found between the standard and both shortage and excess conditions. Standard conditions showed the lowest IA, around 12%, while excess and shortage conditions showed higher inhibition, with values of around 30% and 25%, respectively. For PR, differences were found between the shortage and both excess and standard conditions. Shortage conditions showed the highest IA of around 54%, while excess and standard conditions showed lower inhibition, with inhibitory rates of 30% and 37%, respectively.

#### 3.4.3. Inhibition of the Reproductive Fungal System. Spore Counting

New data were obtained from the same samples as in the section “Isolation and identification of filamentous fungi from contaminated samples”, in this case, related to the action of *D. hansenii* on the production of fungal spores. As described in the Section 2, spore samples from a known area were extracted from solid medium plates. From these counts, two different approaches were used to analyze the data. In the first, the total number of spores produced in the specific extraction area was compared, and in the second, this data was cross-checked with the total mycelial expansion area.

#### 3.4.4. Integrative Analysis of Spore Production Relative to Mycelial Expansion

First, the total spores produced in the total expansion area of the molds were calculated using the formula (MEA × SCE)/EA, where MEA corresponds to the mycelial expansion area, SCE corresponds to spore counts in the extracted agar area, and EA corresponds to the extracted agar area. After that, the IA was calculated by applying [(TSC − TST)/TSC] × 100, where TSC stands for total spore count in the control plates and TST for total spore count in the treated ones. The last formula applied was based on that described in the Section 2.

**Figure 7:**
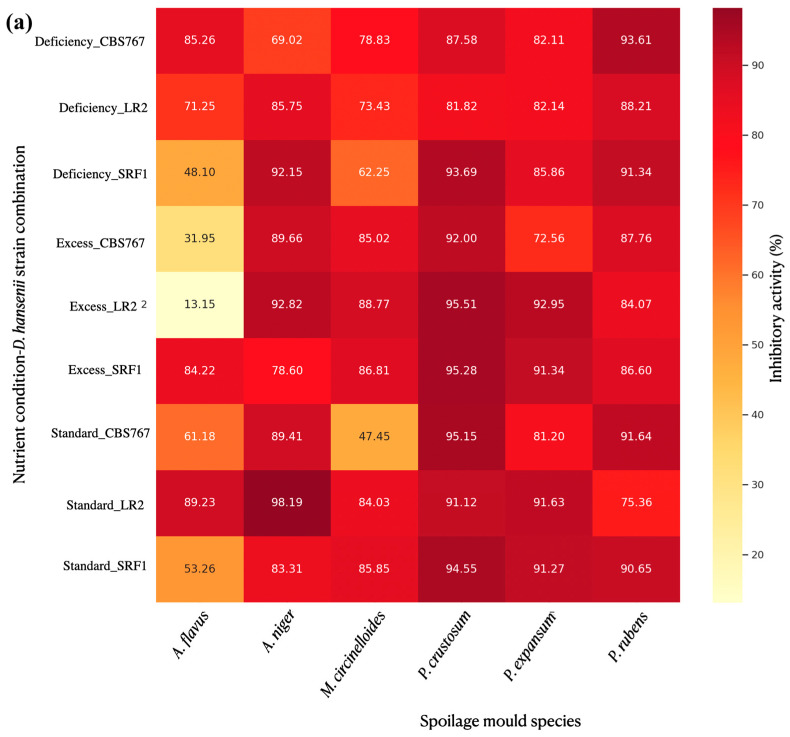

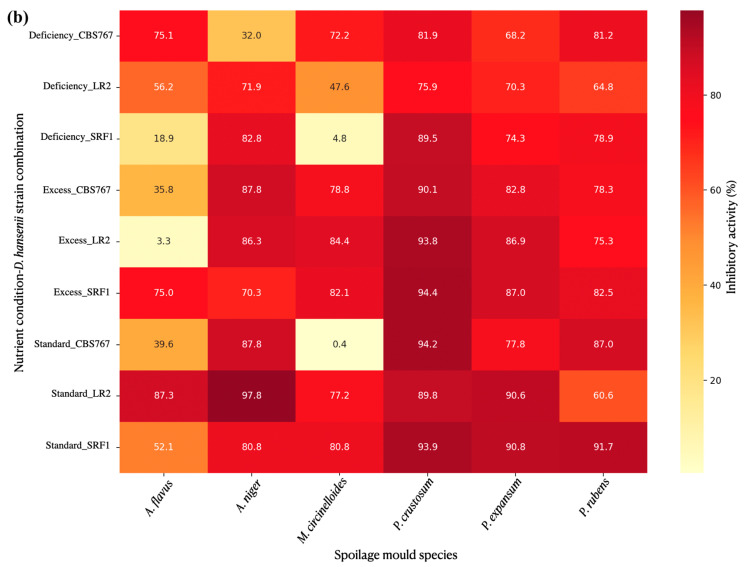
Heatmap plot representations of mean inhibitory activities (IAs) obtained for each studied mold−yeast−nutrient concentration interaction described in the section “Inhibition affection in reproductive fungal system. Spore counting”. (**a**) Represents the results obtained when fungal spore measurements were made considering the total mycelial expansion zones. We observed significant inhibition in most cases, but many more exceptions can be found with respect to the other measurement methods. In (**b**), fungal spore measurements were performed by considering equal production zones. In general, the inhibition percentage was in the 60–100% range, with very few exceptions. Most of these exceptions can be observed for *A. flavus* (AF) mold.

The results showed that, as indicated in the previous section, *D. hansenii* had an influence on the spore production in most of the cases studied (Figure 7), achieving a maximum IA of 97.84% in the case of AN, 94.35% for PC, 91.66% for PR, 91.66% for PE, 90.79% for AF, and the lowest maximum IA among the tested molds, 87.31% for MC. These results slightly differed from those obtained without considering mycelial expansion; therefore, further analysis was performed.

**Figure 8:**
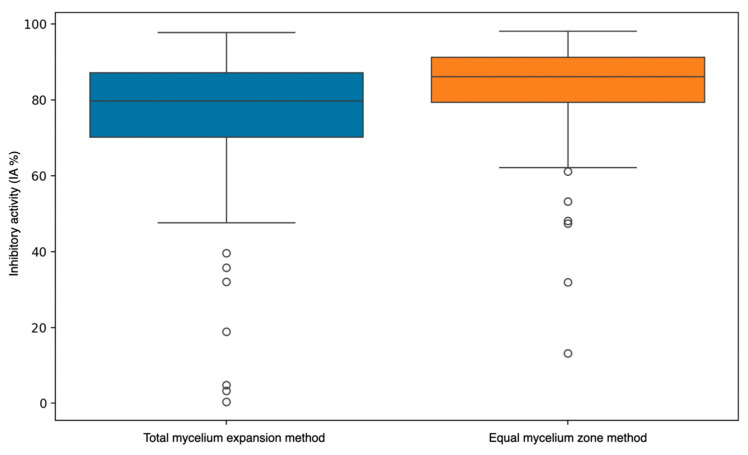
Box plot representation of mean inhibitory activities (IAs) obtained from all the studied mold−yeast−nutrient conditions. It can be observed that data obtained with the method of comparison of equal areas clearly resulted in a lower variability. The control values of IA are not plotted as always correspond to zero value.

The dataset without area consideration generally showed higher IA values and less variability (Figure 8). This suggests that the method of calculation (considering or not considering the total mycelial expansion) has a substantial impact on the reported inhibitory activity of yeasts against molds.

The analysis of the data indicated that while there are some observable differences in inhibitory activity among the nutrient conditions, these differences are not statistically significant based on the ANOVA results, as described in the previous section. Taking that into consideration, a deeper analysis of each of the strains used was performed.

#### 3.4.5. Specific Mold Species Spore Production Affection by *D. hansenii* in Modified Nutrient Concentration Conditions: **Relative to Mycelial Expansion**

For the genera *Aspergillus sp*. and *Mucor* sp. no significant differences were found among the studied conditions and strains. However, it is worth noting that for MC, the difference between shortage and excess conditions is quite large, even though it is not statistically significant.

In the case of *Penicillium sp*., some differences were observed. The analysis revealed statistically significant differences in IA between the shortage and both excess and standard nutrient conditions, indicating that nutrient conditions influence the IA of PE. In this case, nutrient deficiency significantly reduced the inhibitory activity against molds compared to both excess and standard nutrient conditions. When analyzing the PC data, the results showed that the IA was lower in the shortage condition than in the excess and standard conditions. Interestingly, in the PC case, when performing an ANOVA analysis, significant differences were found (Table S3), while when performing a Tukey test, these differences faded (Table S4). These results suggest that while nutrient deficiency may reduce the inhibitory activity against PC, the effect is not as pronounced or clear as for PE. The lack of significant pairwise differences despite a significant ANOVA result indicates that the effect of nutrient conditions on IA for PC is more subtle and may require further investigation or a larger sample size for full characterization. Finally, the PR data analysis did not reveal any statistically significant differences among the conditions in any of the tests performed.

#### 3.4.6. Quantitative Assessment of Spore Production in Defined Extraction Zones

In the first approach, the IA was calculated using the general formula described in Section 2.6. The results showed that *D. hansenii* had an influence on spore production in most of the cases studied (Figure 7b), achieving a maximum IA of 98.19% in the case of AN, 95.51% for PC, 93.61% for PR, 92.95% for PE, 89.23% for AF, and the lowest maximum IA among the tested molds, 88.77% for MC. In general, spore production was significantly affected, with few exceptions. Based on the data and analysis performed comparing the different IA obtained in each of the nutrient concentration conditions studied, the nutrient condition did not appear to significantly influence the IA of the yeasts against the molds in the performed assay.

#### 3.4.7. Specific Mold Species Spore Production Affection by *D. hansenii* Under Modified Nutrient Concentration Conditions: Defined Extraction Zones

In general terms, no statistically significant differences in IA were found in spore production for any of the studied molds. In the *Penicillium* sp. case, even though the results were significant, some tendencies were observed. PC showed the lowest *p*-value, suggesting that, although not statistically significant, there may be a trend toward differences in inhibitory activity across nutrient conditions. PR showed slightly negative mean differences between shortage and other conditions, suggesting that a shortage in nutrients might lead to slightly lower inhibitory activity.

## 4. Discussion

The shift away from chemical preservatives reflects the growing consumer demand for healthier and safer products using natural methods. This trend is supported by scientific research highlighting the potential health risks associated with the excessive use of chemical preservatives [1,2,3]. Yeasts like *Debaryomyces hansenii* have gained interest as biocontrol agents due to their antifungal properties [4,5,6]. Our study explored the antifungal potential of *D. hansenii* in the food industry. Known for its genetic uniqueness and ability to enhance the aroma, texture, and taste of cured meats [17,18], *D. hansenii* also boasts a safety profile validated by its inclusion on the EFSA QPS list. This research highlights new data on the nutrient competition mechanism of *D. hansenii*, enhancing our understanding of its antifungal action. These findings will help deepen our knowledge of this yeast as a natural preservative by better understanding its effects not only on molds but also on the product itself.

Even if nutrient competition is a biocontrol mechanism widely mentioned in the literature, very little work has focused on its actual role and contribution to the whole biocontrol process. One of the first reports on this matter was published in 1989. This reinforces the idea that nutrients have been in the minds of researchers for decades, but even so, until recently, no in-depth studies have been conducted on this subject. That work focused on the nutrient competition between *D. hansenii* and *Penicillium digitatum*, examining the effect of nutrient excess conditions using grapefruit peel as a nutrient solution [35]. Our methodology improves upon this by examining both nutrient excess and shortage conditions, providing a more comprehensive analysis of the inhibitory potential of *D. hansenii*. By testing various nutrient conditions, we gained a deeper understanding of how nutrient availability impacts fungal inhibition, thereby improving the accuracy of our study. Additionally, we utilized sterile cellophane membranes to assess whether yeast nutrient assimilation, in the absence of direct yeast presence, affects fungal growth. This aspect of our study is crucial for determining whether the inhibitory effects of *D. hansenii* persist even after its removal, offering practical insights into its real-world application as a biocontrol agent.

In Figure 6, there are clear signs of fungal inhibition to varying degrees in all nutrient conditions studied. However, when measuring and studying inhibitory activity, as indicated in this work, we concluded that the concentration of nutrients does not seem to play a primary role in this inhibition. Nutrient concentration does impact the overall growth of the plates. The left parts of the plates presented in Figure 6, which grew without the interference of *D. hansenii*, showed lower growth and fewer nutrients in the medium. When we compare IA, we do it proportionally, meaning that each yeast−mold−nutrient condition value obtained was relative to its own control. An illustrative example of this is the mycelial expansion of the *D. hansenii* SFR1 strain under standard nutrient conditions for AF was 0.456 ± 0.010 cm^2^, while the control for that same mold had 0.653 ± 0.003 cm^2^ of mycelial expansion. When applying the formula, this resulted in an IA of 30.07 ± 1.50%. The values for the same calculations, but for nutrient excess conditions, were 0.416 ± 0.004 cm^2^ for treated samples, and 0.623 ± 0.001 cm^2^ for control samples. The resulting IA was 33.10 ± 6.10%. Even though the data are different in each case, when relativizing it to their controls, the significance is not present. Nevertheless, the methodology used takes into consideration mycelial expansion values and relative spore production. Mold behavior is difficult to measure because many factors must be considered, such as the density growth of the mycelium. The methodology used needs further investigation in the future; however, it is the first quantitative approach in this complex type of study.

Much more recently, the carbon source utilization patterns of biocontrol agents, including *D. hansenii* and *P. nordicum*, have been examined [36]. The authors calculated the Niche Overlap Index (NOI) to infer nutrient competition and conducted dual-culture assays to observe direct interactions and potential nutrient and space competition. Our methodology has several complementary aspects. By utilizing sterile cellophane membranes and varying nutrient conditions, we directly assessed fungal inhibition through observable outcomes, such as mycelial growth and spore production. In contrast to the inferred metabolic capabilities and nutrient competition suggested by the NOI method, our approach demonstrated the real-world efficacy of *D. hansenii* in inhibiting fungi under different environmental conditions, providing tangible results. Testing inhibition under conditions of nutrient excess and shortage offers insights into how nutrient availability affects the inhibitory performance of *D. hansenii*, an aspect that has not been addressed before. This adds a practical dimension, demonstrating the potential robustness and adaptability of yeast in various food preservation scenarios. Our findings highlight the importance of both direct interactions and environmental factors in biocontrol research, contributing to a more comprehensive understanding of yeast−mold dynamics.

Molds are a common concern in food safety due to their potential to produce harmful mycotoxins. These toxic compounds pose significant health risks to consumers and lead to economic losses in the food industry. Specific mold genera, such as *Penicillium* and *Aspergillus,* are commonly found in foods like cheese and can produce mycotoxins, such as ochratoxin A, cyclopiazonic acid, and sterigmatocystin [45]. When preservatives, such as nitrites, are reduced, the safety of the product is put at risk, as they play a crucial role in preventing microbial proliferation. When talking about molds, they have a major impact on their growth, primarily through mechanisms involving oxidative stress and cytotoxicity [46]. With that being stated, the results obtained from the fungal isolation performed in this work are not surprising. In conditions of reduced preservatives, mycotoxin-productive species such as *Aspergillus flavus* and *Penicillium crustosum,* among others, have appeared, most of them with known mycotoxin production profiles [42,43,44]. This highlights the importance of finding natural and effective alternatives to nitrites.

In the literature, many works can be found related to the inhibitory potential of *D. hansenii* against several molds [8,20,21,33,34,35]. In this work, various species of *Penicillium* sp., *Aspergillus* sp., and *Mucor* sp. were subjected to inhibition assays against two autochthonous strains of *D. hansenii* and a control strain. Several species of *Penicillium* have been extensively used to study their interactions with *D. hansenii* [10,11,19,33,35,36]. In Núñez et al. [33] cocultivation of *Debaryomyces* and *Penicillium* was performed, they tested up to 39 *D. hansenii* isolates which, in most cases, produced a growth reduction greater than 60% against *P. nordicum*, *P. expansum*, *P. verrucosum*, and *P. commune* strains. Another example of this type of assay is the one performed in a previous work by our group [10], where we also tested *P. expansum* and *P. verrucosum*, obtaining inhibition rates of up to 60%. In this work, we have been able to observe a variety of inhibition rates for *Penicillium* sp. When confronting it directly to our studied yeast strains, *P. rubens* (PR) proved to be more sensitive to *D. hansenii* action, with inhibition rates of up to 75% for the autochthonous strains LR2 and SFR1 and up to 65% for the control strain CBS767. These results agree with previous results available in the literature, indicating that the presence of autochthonous strains of *D. hansenii* is an effective biocontrol agent for this species [10,11,19,33,35,36]. The outcomes for *P. crustosum* (PC) were not as expected. Not much inhibition was detected, but in the case of *D. hansenii* SRF1, the inhibition rate was up to 45%. Although this result is significant, it highlights the need to conduct specific tests by species when seeking biocontrol systems, as the effectiveness of inhibition is directly influenced by the mold–yeast interaction.

The atypical behavior of the *P. crustosum* (PC) mold, showing higher inhibitory activity (IA) in the indirect presence of *D. hansenii* (SL) compared to its direct presence (CL), contrasts with the general trend observed for other molds. This suggests unique interactions between the *P. crustosum* and *D. hansenii* strains. *P. crustosum’s* higher inhibition in SL hints it is more vulnerable to indirect inhibitory mechanisms, as secondary metabolites production than, to direct competition or physical interaction. One possible example is the production of killer toxins related to the affection of the mold cell wall [21,47]. The strain-specific differences, such as the CBS767 strain’s effectiveness in SL, highlight the complexity of yeast−mold interactions and the need for further research into the specific mechanisms involved. Using semipermeable membranes and different nutrient conditions, we observed significant variation in the inhibition rates of PR in terms of mycelial expansion and spore production. Comparing with PC results, differences in mycelial expansion were evident, showing varying degrees of inhibition. However, spore production inhibition by *D. hansenii* did not exhibit these variations and was even higher in some specific cases. Contrasting these findings with the available literature on *P. expansum* (PE), we confirmed that the response to *D. hansenii* was similar to that of PC, validating the sensitivity described in previous works [10,11]. These results underline the complexity of this type of research, where more detailed information is required. Addressing the nutrient concentration differences, new details are brought to the table as our results suggest that adequate nutrition is important for PE to maintain its highest inhibitory potential against molds, and none of the nutritional concentrations tested provided additional benefit over standard conditions for inhibitory activity against PC.

*Aspergillus* species, known for producing harmful mycotoxins, are a significant concern in food products. Similar to *Penicillium*, *Aspergillus* is one of the most widely used molds in biocontrol trials with *D. hansenii*. Medina-Córdova et al. [8] confronted *D. hansenii* against *Aspergillus* sp. in a radial inhibition assay, performed as described in the Section 2 of this work. They observed up to 98% inhibition of mycelial expansion compared to the control without yeast. Delgado et al. [48] also explored the action of *D. hansenii* in *Aspergillus* sp., they used the yeast mixed with *Pseudomonas fluorescens* antifungal protein (PgAFP) applying it on the surface of sliced sausages to inoculate *A. parasiticus* spores afterwards. This resulted in a decrease in *A. parasiticus* counts by about 3 log units compared to untreated samples. If we apply the formula to the available data, we obtain a reduction of around 60% in the spore count. Although the species used in our study are different from those described, and the number of yeast–mold combinations we tested, our results are still aligned with these studies. In terms of mycelial expansion in the presence of yeast, as studied by Medina-Córdova et al. [8], we observed that the autochthonous strains LR2 and SRF1 showed higher inhibition rates against *A. flavus* (AF) compared to the *D. hansenii* control strain CBS767. This suggests that autochthonous strains are more effective as biocontrol agents. When examining spore production data, we found that the inhibition rates for AF and *A. niger* (AN) were higher and more consistent for AN across different nutrient conditions than those for AF. This indicates that AF is more sensitive to nutrient changes than AN, highlighting the importance of species-specific analyses rather than generalizing the results.

In general, *Mucor* species, including *Mucor circinelloides*, are opportunistic pathogens, particularly in immunocompromised hosts, leading to serious infections. There is also evidence of the mycotoxin potential capability of this genus [49]. Medina-Córdova et al. [8] also studied this genus, finding inhibition rates in mycelial expansion up to 98%. Our results showed that in the presence of yeast, the inhibition rates were around 50% for the control strain CBS767 and 90% and 85% for autochthonous strains LR2 and SRF1, respectively, indicating better performance of the autochthonous strains in inhibiting mycelial expansion. Spore production inhibition was consistently around 80%.

Finally, it is important to address the two outcomes presented at the time of comparing spore production. The differences outlined in our results between the two methods could have important implications for interpreting the effectiveness of yeasts in inhibiting mold growth. Researchers should be cautious when comparing results from studies using different calculation methods and should specify whether total mycelial expansion was considered in their IA calculations. By applying the method without the total mycelial area, a comparison is made of the number of spores per specific area, which makes the analysis more realistic as spore production ranges differently depending on the mycelial zone.

## 5. Conclusions

This study demonstrates that *D. hansenii* inhibits mold growth and sporulation. In addition, the autochthonous yeast strains LR2 and SRF1 inhibited mold growth significantly more than the control strain, CBS767. The effectiveness of inhibition varies significantly depending on the specific yeast−mold combination, emphasizing the necessity for case-specific analyses. Generalizing fungal genus behavior in this context is inadvisable due to the numerous influencing factors that could compromise accuracy. Specific interactions and conditions must be considered to draw reliable conclusions. Comparing spore data per area is essential for realistic analysis. *D. hansenii* affects both mycelial expansion and spore production in the molds studied. The direct presence of yeast plays a significant role in inhibition, highlighting the need for a deeper analysis of yeast-cell interactions and metabolite production. This work is the first clear-cut example of the real importance of nutrient competition in the biocontrol capacity of *D. hansenii*. Nutrient competition generally does not directly impact overall inhibition, relegating this mechanism, which is widely accepted to hold significant importance, to a complementary role. Ultimately, our methodology reaffirms the inhibitory potential of *D. hansenii*, highlighting its versatility and resilience. This advances food safety and preservation by providing new insights into effective natural biocontrol methods.

## Figures and Tables

**Table 1:** Preservative conditions (mg/kg) of the samples studied. Table salt (NaCl) was used as the salt. In the column isolates, relative information regarding the percentage of presence of each type of microorganism in relation to the total, considering 100% as the complete set of that type of microorganism isolated under all conditions.

NaCl (Table Salt)	Nitrites	Condition Name	Reduction Percentage
25	5.0	25/5.0	0% (Commercial sample)
25	4.5	25/4.5	10%
25	4.0	25/4.0	20%
25	3.5	25/3.5	30%
20	5.0	20/5.0	20%

**Table 2:** Identification of microorganisms using Sanger sequencing.

Isolate	Identification	Similarity
HSC30	*Penicillium crustosum*	100.00%
HPDA3	*Penicillium echinulatum*	100.00%
HPDA10	*Penicillium rubens*	100.00%
HSC34	*Mucor racemosus*	100.00%
HPDA14	*Penicillium echinulatum*	100.00%
HPDA7	*Penicillium crustosum*	100.00%
HYPD3	*Mucor circinelloides f. circinelloides*	100.00%
HSC31	*Aspergillus flavus*	99.46%

**Table 3:** Percentage of presence of isolated species for each type of microorganism (bacteria, yeast, or mold), considering 100% as the complete set of that type of microorganism. CL stands for yeast-inoculated samples and SL for non-inoculated samples.

Condition Name	Presence Percentage (%)					
	Bacteria		Yeast		Mold	
	CL	SL	CL	SL	CL	SL
25/5.0	45.00	50.00	50.00	62.50	57.14	57.14
25/4.5	40.00	50.00	37.50	37.50	71.43	71.43
25/4.0	50.00	60.00	62.50	50.00	71.43	71.43
25/3.5	55.00	55.00	50.00	50.00	85.71	85.71
20/5.0	65.00	60.00	50.00	50.00	42.86	71.43

## Data Availability

Data will be made available on request.

## Supplementary Materials

The following supporting information can be downloaded at: https://www.mdpi.com/article/10.3390/jof11040242/s1, Table S1: One-way ANOVA results of the general comparison between nutrient concentration conditions (excess, standard and deficiency); Table S2: Multiple comparison of means (Tukey) post-hoc test results for the comparison between nutrient concentration conditions (excess, standard and deficiency) groups; Table S3: One-way ANOVA results of the general comparison between nutrient concentration conditions (excess, standard and deficiency) for spore production affection of *P. crustosum* by *D. hansenii* in modified nutrient concentration conditions (relative to mycelial expansion); Table S4: Multiple comparison of means (Tukey) post-hoc test results for the comparison between nutrient concentration conditions (excess, standard and deficiency) groups for spore production affection of *P. crustosum* by *D. hansenii* in modified nutrient concentration conditions (relative to mycelial expansion); Table S5: One-way ANOVA results of the general comparison between nutrient concentration conditions (excess, standard and deficiency) for spore production affection of *P. rubens* by *D. hansenii* in modified nutrient concentration conditions (relative to mycelial expansion); Table S6: Multiple comparison of means (Tukey) post-hoc test results for the comparison between nutrient concentration conditions (excess, standard and deficiency) groups for spore production affection of *P. rubens* by *D. hansenii* in modified nutrient concentration conditions (relative to mycelial expansion).

## References

[1] Van Loo E.J., Ricke S.C., O’bryan C.A., Johnson M.G., Ricke S.C., Van Loo E.J., Johnson M.G., O’Bryan C.A. (2012). Historical and Current Perspectives on Organic Meat Production. Organic Meat Production and Processing.

[2] Gokoglu N. (2019). Novel Natural Food Preservatives and Applications in Seafood Preservation: A Review. J. Sci. Food Agric..

[3] Novais C., Molina A.K., Abreu R.M.V., Santo-Buelga C., Ferreira I.C.F.R., Pereira C., Barros L. (2022). Natural Food Colorants and Preservatives: A Review, a Demand, and a Challenge. J. Agric. Food Chem..

[4] Avis T.J., Belanger R.R. (2002). Mechanisms and Means of Detection of Biocontrol Activity of *Pseudozyma* Yeasts against Plant-Pathogenic Fungi. FEMS Yeast Res..

[5] Liu J., Sui Y., Wisniewski M., Droby S., Liu Y. (2013). Review: Utilization of Antagonistic Yeasts to Manage Postharvest Fungal Diseases of Fruit. Int. J. Food Microbiol..

[6] Sipiczki M. (2023). Identification of Antagonistic Yeasts as Potential Biocontrol Agents: Diverse Criteria and Strategies. Int. J. Food Microbiol..

[7] Agirman B., Carsanba E., Settanni L., Erten H. (2023). Exploring Yeast-based Microbial Interactions: The next Frontier in Postharvest Biocontrol. Yeast.

[8] Medina-Córdova N., López-Aguilar R., Ascencio F., Castellanos T., Campa-Córdova A.I., Angulo C. (2016). Biocontrol Activity of the Marine Yeast *Debaryomyces hansenii* against Phytopathogenic Fungi and Its Ability to Inhibit Mycotoxins Production in Maize Grain (*Zea mays* L.). Biol. Control.

[9] Shwaiki L.N., Arendt E.K., Lynch K.M., Thery T.L.C. (2019). Inhibitory Effect of Four Novel Synthetic Peptides on Food Spoilage Yeasts. Int. J. Food Microbiol..

[10] Chacón-Navarrete H., Ruiz-Pérez F., Ruiz-Castilla F.J., Ramos J. (2022). Exploring Biocontrol of Unwanted Fungi by Autochthonous *Debaryomyces hansenii* Strains Isolated from Dry Meat Products. J. Fungi.

[11] Chacón-Navarrete H., Gómez M., Cardador M.J., Salatti-Dorado J.Á., Pérez-Cacho P.R., Roldán-Casas J.Á., Arce L., Galán-Soldevilla H., López B., Ramos J. (2024). The Antimycotic Potential of *Debaryomyces hansenii* LRC2 on Iberian Pork Loins with Low Concentration Preservatives. Food Control.

[12] Butler G., Rasmussen M.D., Lin M.F., Santos M.A.S., Sakthikumar S., Munro C.A., Rheinbay E., Grabherr M., Forche A., Reedy J.L. (2009). Evolution of Pathogenicity and Sexual Reproduction in Eight *Candida* Genomes. Nature.

[13] Gancedo C., Serrano R. (1989). Energy-Yielding Metabolism.

[14] Sugita T., Nakase T. (1999). Non-Universal Usage of the Leucine CUG Codon and the Molecular Phylogeny of the Genus *Candida*. Syst. Appl. Microbiol..

[15] Dujon B., Sherman D., Fischer G., Durrens P., Casaregola S., Lafontaine I., De Montigny J., Marck C., Neuvéglise C., Talla E. (2004). Genome Evolution in Yeasts. Nature.

[16] Breuer U., Harms H. (2006). *Debaryomyces Hansenii*—An Extremophilic Yeast with Biotechnological Potential. Yeast.

[17] Aquilanti L., Santarelli S., Silvestri G., Osimani A., Petruzzelli A., Clementi F. (2007). The Microbial Ecology of a Typical Italian Salami during Its Natural Fermentation. Int. J. Food Microbiol..

[18] Prista C., Michán C., Miranda I.M., Ramos J. (2016). The Halotolerant *Debaryomyces hansenii*, the Cinderella of Non-conventional Yeasts. Yeast.

[19] Virgili R., Simoncini N., Toscani T., Camardo Leggieri M., Formenti S., Battilani P. (2012). Biocontrol of *Penicillium nordicum* Growth and Ochratoxin A Production by Native Yeasts of Dry Cured Ham. Toxins.

[20] Andrade M.J., Thorsen L., Rodríguez A., Córdoba J.J., Jespersen L. (2014). Inhibition of Ochratoxigenic Moulds by *Debaryomyces hansenii* Strains for Biopreservation of Dry-Cured Meat Products. Int. J. Food Microbiol..

[21] Çorbacı C., Uçar F.B. (2018). Purification, Characterization and in Vivo Biocontrol Efficiency of Killer Toxins from *Debaryomyces hansenii* Strains. Int. J. Biol. Macromol..

[22] Spadaro D., Droby S. (2016). Development of Biocontrol Products for Postharvest Diseases of Fruit: The Importance of Elucidating the Mechanisms of Action of Yeast Antagonists. Trends Food Sci. Technol..

[23] Freimoser F.M., Rueda-Mejia M.P., Tilocca B., Migheli Q. (2019). Biocontrol Yeasts: Mechanisms and Applications. World J. Microbiol. Biotechnol..

[24] Simoncini N., Virgili R., Spadola G., Battilani P. (2014). Autochthonous Yeasts as Potential Biocontrol Agents in Dry-Cured Meat Products. Food Control.

[25] Pleadin J., Frece J., Markov K. (2019). Mycotoxins in Food and Feed. Advances in Food and Nutrition Research.

[26] Ritz K. (1995). Growth Responses of Some Soil Fungi to Spatially Heterogeneous Nutrients. FEMS Microbiol. Ecol..

[27] Crowther T.W., Boddy L., Jones T.H. (2011). Outcomes of Fungal Interactions Are Determined by Soil Invertebrate Grazers: Grazers Alter Fungal Community. Ecol. Lett..

[28] Choudhury M., Trevelyan P., Boswell G.A. (2018). Mathematical Model of Nutrient Influence on Fungal Competition. J. Theor. Biol..

[29] Guo Y., He Y., Wu P., Wu B., Lin Y., He M., Han X., Xia T., Shen K., Kang L. (2022). The Interspecific Competition Presents Greater Nutrient Facilitation Compared with Intraspecific Competition through AM Fungi Interacting with Litter for Two Host Plants in Karst Soil. J. Plant Ecol..

[30] Hilber-Bodmer M., Schmid M., Ahrens C.H., Freimoser F.M. (2017). Competition Assays and Physiological Experiments of Soil and Phyllosphere Yeasts Identify *Candida subhashii* as a Novel Antagonist of Filamentous Fungi. BMC Microbiol..

[31] Ramos-Moreno L., Ruiz-Castilla F.J., Bravo C., Martínez E., Menéndez M., Dios-Palomares R., Ramos J. (2019). Inoculation with a Terroir Selected *Debaryomyces Hansenii* Strain Changes Physico-Chemical Characteristics of Iberian Cured Pork Loin. Meat Sci..

[32] Rivas-Garcia T., Murillo-Amador B., Nieto-Garibay A., Rincon-Enriquez G., Chiquito-Contreras R.G., Hernandez-Montiel L.G. (2019). Enhanced Biocontrol of Fruit Rot on Muskmelon by Combination Treatment with Marine *Debaryomyces hansenii* and *Stenotrophomonas rhizophila* and Their Potential Modes of Action. Postharvest Biol. Technol..

[33] Núñez F., Lara M.S., Peromingo B., Delgado J., Sánchez-Montero L., Andrade M.J. (2015). Selection and Evaluation of *Debaryomyces hansenii* Isolates as Potential Bioprotective Agents against Toxigenic Penicillia in Dry-Fermented Sausages. Food Microbiol..

[34] Hernandez-Montiel L.G., Gutierrez-Perez E.D., Murillo-Amador B., Vero S., Chiquito-Contreras R.G., Rincon-Enriquez G. (2018). Mechanisms Employed by *Debaryomyces hansenii* in Biological Control of Anthracnose Disease on Papaya Fruit. Postharvest Biol. Technol..

[35] Droby S., Chalutz E., Wilson C.L., Wisniewski M. (1989). Characterization of the Biocontrol Activity of *Debaryomyces hansenii* in the Control of *Penicillium Digitatum* on Grapefruit. Can. J. Microbiol..

[36] Álvarez M., Núñez F., Delgado J., Andrade M.J., Rodríguez M., Rodríguez A. (2021). Competitiveness of Three Biocontrol Candidates against Ochratoxigenic *Penicillium nordicum* under Dry-Cured Meat Environmental and Nutritional Conditions. Fungal Biol..

[37] Ramos J., Melero Y., Ramos-Moreno L., Michán C., Cabezas L. (2017). *Debaryomyces Hansenii* Strains from Valle De Los Pedroches Iberian Dry Meat Products: Isolation, Identification, Characterization, and Selection for Starter Cultures. J. Microbiol. Biotechnol..

[38] Prista C., Almagro A., Loureiro-Dias M.C., Ramos J. (1997). Physiological Basis for the High Salt Tolerance of *Debaryomyces hansenii*. Appl. Environ. Microbiol..

[39] Conlon B.H., Schmidt S., Shik J.Z. (2022). Orthogonal Protocols for DNA Extraction from Filamentous Fungi. STAR Protoc..

[40] Usyk M., Zolnik C.P., Patel H., Levi M.H., Burk R.D. (2017). Novel ITS1 Fungal Primers for Characterization of the Mycobiome. mSphere.

[41] Velle L. (2002). Osmosis. J. Biol. Edu..

[42] Rundberget T., Skaar I., Flåøyen A. (2004). The Presence of *Penicillium* and *Penicillium* Mycotoxins in Food Wastes. Int. J. Food Microbiol..

[43] Snyder A.B., Churey J.J., Worobo R.W. (2016). Characterization and Control of *Mucor circinelloides* Spoilage in Yogurt. Int. J. Food Microbiol..

[44] Tai B., Chang J., Liu Y., Xing F. (2020). Recent Progress of the Effect of Environmental Factors on *Aspergillus flavus* Growth and Aflatoxins Production on Foods. Food Qual. Saf..

[45] Kure C.F., Skaar I. (2019). The Fungal Problem in Cheese Industry. Curr. Opin. Food Sci..

[46] Patsoukis N., Georgiou C.D. (2007). Effect of Sulfite–Hydrosulfite and Nitrite on Thiol Redox State, Oxidative Stress and Sclerotial Differentiation of Filamentous Phytopathogenic Fungi. Pestic. Biochem. Physiol..

[47] Al-Qaysi S.A.S., Al-Haideri H., Thabit Z.A., Al-Kubaisy W.H.A.A., Ibrahim J.A.A. (2017). Production, Characterization, and Antimicrobial Activity of Mycocin Produced by *Debaryomyces hansenii* DSMZ70238. Int. J. Microbiol..

[48] Delgado J., Rodríguez A., García A., Núñez F., Asensio M.A. (2018). Inhibitory Effect of PgAFP and Protective Cultures on *Aspergillus parasiticus* Growth and Aflatoxins Production on Dry-Fermented Sausage and Cheese. Microorganisms.

[49] Lee S.C., Billmyre R.B., Li A., Carson S., Sykes S.M., Huh E.Y., Mieczkowski P., Ko D.C., Cuomo C.A., Heitman J. (2014). Analysis of a Food-Borne Fungal Pathogen Outbreak: Virulence and Genome of a *Mucor circinelloides* Isolate from Yogurt. mBio.

