# Supplementary material for: Evaluating the Role of Nutrient Competition in Debaryomyces hansenii Biocontrol Activity Against Spoilage Molds in the Meat Industry
Source: J Fungi (Basel). 2025 Mar 22;11(4):242. doi: 10.3390/jof11040242 (PMC12027869; doi:10.3390/jof11040242)
Supplement: Supplementary file 1 [file jof-11-00242-s001.zip › jof-3519345-supplementary.pdf]

Table S1. One-way ANOVA results of the general comparison between nutrient concentration conditions (excess, standard and deficiency).

Source of Variation	Sum of Squares	df	F	p-value
Between Groups	2472.498	2		
Within Groups	21987.507	159	8.9398	0.00021
Total	24460.005			

Table S2. Multiple comparison of means (Tukey) post-hoc test results for the comparison between nutrient concentration conditions (excess, standard and deficiency) groups.

Group comparison	Mean difference	Adjusted p-value	Lower Confidence Limit	Upper Confidence Limit	Significance
Deficiency *vs.* excess	-2.3461	0.5549	-7.7003	3.0082	True
Deficiency *vs.* standard	-9.2075	0.0002	-14.5617	-3.8532	False
Excess *vs.* standard	-6.8614	0.0079	-12.2157	-1.5072	False

Table S3. One-way ANOVA results of the general comparison between nutrient concentration conditions (excess, standard and deficiency) for spore production affection of *P. crustosum* by *D. hansenii* in modified nutrient concentration conditions (relative to mycelial expansion).

Source of Variation	Sum of Squares	df	F	p-value
Between Groups	210.5422	2		
Within Groups	115.4423	6	5.4714	0.0444
Total	325.9845			

Table S4. Multiple comparison of means (Tukey) post-hoc test results for the comparison between nutrient concentration conditions (excess, standard and deficiency) groups for spore production affection of *P. crustosum* by *D. hansenii* in modified nutrient concentration conditions (relative to mycelial expansion).

Group comparison	Mean difference	Adjusted p-value	Lower Confidence Limit	Upper Confidence Limit	Significance
Deficiency *vs.* excess	10.3039	0.0634	-0.685	21.2929	False
Deficiency *vs.* standard	10.2158	0.0654	-0.7731	21.2048	False
Excess *vs.* standard	-0.0881	0.9997	-11.077	10.9008	False

Table S5. One-way ANOVA results of the general comparison between nutrient concentration conditions (excess, standard and deficiency) for spore production affection of *P. rubens* by *D. hansenii* in modified nutrient concentration conditions (relative to mycelial expansion).

**Source of Variation**	**Sum of Squares**	**df**	**F**	**p-value**
Between Groups	37.4439	2		
Within Groups	745.6001	6	0.1506	0.8632
Total	780.0440			

Table S6. Multiple comparison of means (Tukey) post-hoc test results for the comparison between nutrient concentration conditions (excess, standard and deficiency) groups for spore production affection of *P. rubens* by *D. hansenii* in modified nutrient concentration conditions (relative to mycelial expansion).

Group comparison	Mean difference	Adjusted p-value	Lower Confidence Limit	Upper Confidence Limit	Significance
Deficiency *vs.* excess	3.7136	0.9136	-24.2135	31.6407	False
Deficiency *vs.* standard	4.7514	0.8637	-23.1757	32.6785	False
Excess *vs.* standard	1.0379	0.9929	-26.8892	28.965	False
